# A rare cause of dilated cardiomyopathy: hypocalcemia

**DOI:** 10.20945/2359-3997000000474

**Published:** 2022-05-12

**Authors:** Ummu Mutlu, Ramazan Cakmak, Mehmet Rasih Sonsöz, Ekrem Bilal Karaayvaz, Ayse Kubat Uzum, Refik Tanakol, Ferihan Aral

**Affiliations:** 1 Istanbul University Istanbul Faculty of Medicine Department of Endocrinology and Metabolism Istanbul Turkey Istanbul University, Istanbul Faculty of Medicine, Department of Endocrinology and Metabolism, Istanbul, Turkey; 2 Istanbul University Istanbul Faculty of Medicine Department of Cardiology Istanbul Turkey Istanbul University, Istanbul Faculty of Medicine, Department of Cardiology, Istanbul, Turkey

## Abstract

Dilated cardiomyopathy (DCM) is characterized by systolic dysfunction and is usually idiopathic. A rare cause of reversible DCM is hypocalcemia. Calcium plays a key role in myocardial contraction. Hypocalcemia can lead to a decrease in contraction, left ventricular systolic dysfunction, and heart failure with reduced ejection fraction (EF). Hypocalcemia-related reversible DCM reports are rare. Herein, we present two cases with heart failure caused by hypocalcemia developed due to hypoparathyroidism. The first case presented with severe heart failure and an extremely low serum calcium level (4.4 mg/dL) due to idiopathic hypoparathyroidism. The second case, which was also admitted with heart failure due to hypocalcemia, had iatrogenic hypoparathyroidism due to a subtotal thyroidectomy. In both cases, patients had reduced left ventricular systolic functions (EF was 33% and 42%, respectively). After calcium replacement and heart failure treatment, calcium levels were normalized. A significant and rapid improvement in heart failure was achieved in both cases (EF 60% and 50%, respectively). Serum calcium levels should always be measured in patients with heart failure, and the etiology of hypocalcemia should be sought. In addition to the standard pharmacotherapy of heart failure with reduced EF, calcium supplementation is essential for treating these patients.

## INTRODUCTION

Dilated cardiomyopathy (DCM) is characterized by systolic dysfunction, which develops due to deterioration of the heart muscle's contractility. There may be various diseases in etiology, but sometimes no cause could be found and, in this case, it is referred to as idiopathic DCM (1). Calcium levels are vital because the sarcoplasmic reticulum cannot provide enough calcium for myocardium contraction. Intracellular calcium initiates the contraction cycle by binding to the thin filament regulatory protein troponin C to relieve the inhibition exerted by the troponin complex. As relatively small amounts of Ca^2+^ enter and leave the cardiomyocyte during each cardiac cycle, larger amounts move in and out of the sarcoplasmic reticulum (2). Entry of Ca^2+^ via the voltage-sensitive L-type Ca^2+^ channels act as a trigger for the release of Ca^2+^ from the sarcoplasmic reticulum, which may be adversely affected in hypocalcemia. As a result, contractility may be attenuated, and left ventricular systolic dysfunction may occur (2). Hypocalcemia can lead to a decrease in contraction and DCM (3). Cases of reversible cardiomyopathy due to hypocalcemia have been reported in the literature (4-6). Herein, we present two cases with heart failure caused by hypocalcemia developed due to hypoparathyroidism.

## CASE REPORTS

Case 1: A 41-year-old woman was admitted to the emergency room with leg swelling, shortness of breath, numbness, and hand cramps. The calcium level was 4.4 mg/dL, and the patient was treated with intravenous calcium and diuretics. She was hospitalized for treatment and further evaluation. Medical history revealed bronchial asthma, bilateral cataract surgery, and a right ankle fracture.

Physical examination findings were a weight of 103 kilograms, a height of 155 centimeters, dry skin, fragile nails, teeth hypoplasia, a positive Chvostek sign, blood pressure of 143/78 mmHg, a positive S3, generalized edema, fine inspiratory crackles bilaterally in lungs until middle zones, eczematous lesions on her skin, and alopecia totalis. Laboratory results showed microcytic anemia; creatinine, 0.6 mg/dL; albumin, 3.27 g/dL; calcium, 5.9 mg/dL; phosphorus, 4.3 mg/dL; LDH, 817 U/L; CK, 869 U/L; TSH, 2.9 mIU/L; fT4, 19.5 pmol/L; anti-TPO, 200 IU/mL; PTH, 12.7 pg/mL,; HbAa1c, 7.4%; c-peptide, 5.03 ng/mL; microalbumin/creatinine, 10.6 mg/g; pro-BNP, 1704 pg/mL; and hsTroponin T, 10.66 pg/mL (< 14) (Table 1).

**Tabla 1:** Laboratory results of the patients

	Case 1		Case 2	
	Pre-treatment	Post-treatment 1^st^ year	Pre-treatment	Post-treatment 1^st^ year
Creatinine mg/dL (0,7-1,4)	0,6	0,8	0,7	0,7
Albumin g/dL (3,2-5,5)	3,27	4,7	3,85	4,2
Calcium mg/dL (8,5-10,5)	5,9	8,6	6,1	8,1
Phosphorus mg/dL (2,7-4,5)	4,3	4,7	6,25	4,94
Magnesium mmol/L (0,8-1,2)	0,57	0,70	0,6	0,74
Alkaline phosphatase U/L (35-105)	111	104	120	66
25-OH-vit D3 ng/mL (20-50)	5,7	31	8	11
CK U/L (30-220)	869	116	269	51
PTH pg/mL (15-65)	12,7	11.5	12	7,5
QT interval in ECG ms	560	440	508	438
Ejection fraction %	33	60	42	50

CK: creatin kinase, PTH: parathyroid hormone.

**Figure 1:**
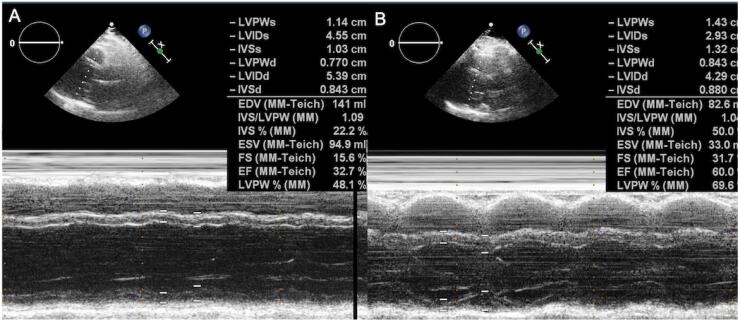
Changes in the pre-treatment (**A**) and post-treatment (**B**) left ventricle function in M-mode echocardiography.

An electrocardiogram (ECG) showed sinus tachycardia. A transthoracic echocardiogram (TTE) revealed reduced left ventricular systolic dysfunction (ejection fraction [EF] was measured as 33% using Teichholz's method) and global left ventricular hypokinesis (Figure 1 A and B). All heart chambers were a normal size.

Calcium, cholecalciferol, magnesium, and calcitriol for hypoparathyroidism and intravenous furosemide for generalized edema were initiated. After the fifth day, the patient's weight was 88 kg; her edema decreased; and calcium was 9.3 mg/dL. Ramipril and carvedilol were started for heart failure.

Further investigation for autoimmune polyglandular syndromes showed no features. Therefore, the patient was diagnosed with idiopathic hypoparathyroidism. A spine X-ray revealed paravertebral ligament calcification. There was no evidence of nephrolithiasis, nephrocalcinosis, or basal ganglia calcifications. The patient's bronchial obstruction ameliorated after calcium replacement.

She was discharged from the hospital with calcium 2,000 mg/daily, calcitriol 1 mcg/daily, magnesium 365 mg/daily, and cholecalciferol 1,600 IU/daily for the treatment of hypoparathyroidism. The follow-up evaluation showed normocalcemia and normophosphatemia with pro-BNP 300 pg/mL (Table 1). After a year, TTE disclosed normal left ventricular systolic function. EF was measured as 60% using Teichholz's method.

Case 2: A 58-year-old woman was admitted to the emergency department with swelling in the legs, shortness of breath (NYHA class III), numbness and cramping in the hands, and around the mouth. Her medical history revealed iatrogenic hypoparathyroidism due to a subtotal thyroidectomy (45-years-ago) and bilateral cataract surgery (15-years-ago). She took an oral calcium replacement. However, in recent years, her treatment has been erratic, and she was admitted to emergency departments periodically for contraction complaints and to receive IV calcium replacement therapy. Recently, she was diagnosed with heart failure.

Physical examination findings were a weight of 120 kg, a height of 158 cm, positive Chvostek and Trousseau signs, and carpopedal spasms in both hands. Moreover, her blood pressure was 143/78 mmHg, with a heart rate of 101 beats per minute, pitting (+) pretibial edema, and fine inspiratory crackles bilaterally in lungs.

An electrocardiogram showed sinus rhythm, a right bundle branch block, and QT interval was 508 ms, corrected for heart rate due to Bazett's formula (7). Laboratory results showed microcytic anemia; creatinine, 0.7 mg/dL; albumin, 3.85 g/dL; calcium, 6.1 mg/dL; phosphorus, 6.25 mg/dL; LDH, 323 U/L; CK, 269 U/L; TSH, 3.2 mIU/L; fT4, 16.1 pmol/L; PTH, 12.7 pg/mL; pro-BNP, 1,022 pg/mL; and high-sensitive troponin T, 14 pg/mL (Table 1).

Parenteral calcium was started for symptomatic hypocalcemia. After the symptoms resolved, treatment was changed to oral calcium 3000 mg/daily, cholecalciferol 2400 IU/daily, magnesium 365 mg/daily, and 1 mcg calcitriol/daily. She was given a phosphorus-restricted diet and phosphorus-binding agents for the hyperphosphatemia.

TTE disclosed moderately reduced left ventricular systolic dysfunction (EF was measured as 42% using a biplane Simpson's method), global left ventricular hypokinesis, left atrial dilatation, grade II mitral regurgitation (secondary to annular dilatation), mild tricuspid regurgitation, and pulmonary hypertension (an estimated pulmonary artery systolic pressure of 37 mmHg). Ramipril 5 mg/daily, carvedilol 6.25 mg/daily, and intravenous furosemide were initiated. Her dyspnea and findings of congestion resolved within days after the normalization of calcium levels. ECG showed sinus rhythm and a corrected QT interval of 438 ms (Table 1). No nephrolithiasis or nephrocalcinosis was detected in a urinary system ultrasound. Basal ganglia calcifications were seen in cranial computerized tomography.

**Figure 2:**
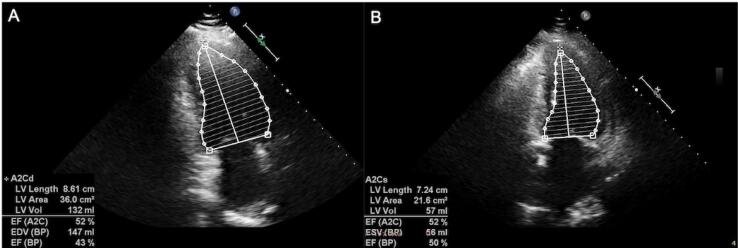
Changes in the pre-treatment (**A**) and post-treatment (**B**) left ventricular ejection fraction measured by biplane Simpson's method.

Control TTE revealed left ventricular systolic function at the lower limit of normal (EF was measured as 50% using a biplane Simpson's method), mild mitral regurgitation, trace tricuspid regurgitation, and an estimated pulmonary artery systolic pressure of 25 mmHg (Figures 2, A, B).

## DISCUSSION

This report of two cases with hypoparathyroidism illustrates one of the few reversible causes of heart failure. Both cases had profound symptoms and signs of congestion, and the patients recovered shortly after a combination of calcium replacement and heart failure therapy.

Dilated cardiomyopathy is a condition characterized by systolic dysfunction and can be reversible caused by, for example, alcoholism, postpartum cardiomyopathy, and hypothyroidism. A cause of reversible DCM is hypocalcemia due to hypoparathyroidism. Hypocalcemia-related reversible DCM reports are rare (8).

Parathyroid hormone (PTH) regulates calcium metabolism through several routes, including the bone resorption by osteoclast activation, the renal absorption of calcium in distal tubule, stimulation of 1-α hydroxylase, and the inhibition of proximal tubular transport of phosphates. PTH is important for calcium balance and homeostasis. After all, PTH deficiency results in hypocalcemia (9). The most common cause of persistent hypoparathyroidism iatrogenic hypoparathyroidism that develops after neck surgery. Other etiologies include irradiation therapies, autoimmune hypoparathyroidism, and genetic mutations (10).

Intracellular calcium plays a key role in the cardiac muscle's systolic and diastolic functions. Chronic and severe hypocalcemia can lead to a decrease in myocardial contractibility and may lead to left ventricular systolic dysfunction and heart failure with reduced EF (3). Recently, several cases of DCM secondary to hypocalcemia have been reported. Overall, the prognosis was good in these cases, and systolic functions returned to normal in 3 to 12 months (11).

In our report, we presented two cases of heart failure secondary to hypocalcemia. The cause was idiopathic hypoparathyroidism in the first case and iatrogenic hypoparathyroidism due to previous thyroid surgery in the second case. In both cases, myocardial contractility was impaired, and pro-BNP levels were elevated, consistent with the findings of heart failure. We presumed that DCM developed because of hypocalcemia, which was not treated for a long time. After a combination of heart failure therapy and calcium replacement, left ventricular systolic functions recovered in both patients, and pro-BNP levels decreased within days.

Patients with chronic hypocalcemia may have subjective symptoms, which are not specific to hypocalcemia, such as chronic fatigue and muscle weakness. It may be difficult to diagnose hypocalcemia in the absence of tetani. Both cases that we presented had a history of cataract surgery. It is well known that hypoparathyroidism is an essential cause of lenticular cataract, especially in younger patients. Ophthalmologists should be aware of metabolic disorders accompanied by cataract, so that some cases can be diagnosed earlier.

Sometimes the patient's initial presentation can be due to heart failure. Diuretic treatment could be started without knowing the serum calcium levels. Specifically, furosemide could decrease the serum calcium level by increasing the calcium excretion in urine, which can lead to worsening symptoms. Hypocalcemia-induced heart failure is resistant to diuretic treatment but rapidly responds to calcium replacement and normalization of the serum calcium levels (12).

Acute coronary syndrome should be a differential diagnosis, as newly diagnosed heart failure with reduced EF may be the first presentation of the myocardial infaction (13). Diabetic patients may not have chest pain due to diabetic neuropathy (14). The dynamic ECG ST-T segment changes, newly diagnosed left ventricular segmental wall motion abnormality, and typical rise and/or fall of cardiac injury markers should prompt this diagnosis (15). In our report, neither case had these features. Besides, the improvement of the left ventricular EF after calcium replacement therapy was considered a reversible cause of heart failure rather than a new myocardial infarction.

In conclusion, hypocalcemia is a reversible cause of heart failure. Serum calcium levels should always be measured in patients with heart failure, and the etiology of hypocalcemia should be sought. In addition to the standard pharmacotherapy of heart failure with reduced EF, calcium supplementation is essential for treating these patients.
